# Deep learning–based integration of genetics with registry data for stratification of schizophrenia and depression

**DOI:** 10.1126/sciadv.abi7293

**Published:** 2022-06-29

**Authors:** Rosa Lundbye Allesøe, Ron Nudel, Wesley K. Thompson, Yunpeng Wang, Merete Nordentoft, Anders D. Børglum, David M. Hougaard, Thomas Werge, Simon Rasmussen, Michael Eriksen Benros

**Affiliations:** 1 Copenhagen Research Centre for Mental Health, Mental Health Centre Copenhagen, Copenhagen University Hospital, Copenhagen, Denmark.; 2 Novo Nordisk Foundation Center for Protein Research, Faculty of Health and Medical Sciences, University of Copenhagen, Copenhagen, Denmark.; 3 iPSYCH, The Lundbeck Foundation Initiative for Integrative Psychiatric Research, Aarhus, Denmark.; 4 Institute of Biological Psychiatry, Mental Health Centre Sct. Hans, Mental Health Services Copenhagen, Roskilde, Denmark.; 5 Herbert Wertheim School of Public Health and Human Longevity Science, University of California, San Diego, San Diego, CA, USA.; 6 Lifespan Changes in Brain and Cognition (LCBC), Department of Psychology, University of Oslo, Forskningsveien 3A, 0317 Oslo, Norway.; 7 Department of Clinical Medicine, Faculty of Health and Medical Sciences, University of Copenhagen, Copenhagen, Denmark.; 8 Department of Biomedicine, Aarhus University and Centre for Integrative Sequencing, iSEQ, Aarhus, Denmark.; 9 Aarhus Genome Center, Aarhus, Denmark.; 10 Center for Neonatal Screening, Department for Congenital Disorders, Statens Serum Institut, Copenhagen, Denmark.; 11 Department of Immunology and Microbiology, Faculty of Health and Medical Sciences, University of Copenhagen, Copenhagen, Denmark.

## Abstract

Currently, psychiatric diagnoses are, in contrast to most other medical fields, based on subjective symptoms and observable signs and call for new and improved diagnostics to provide the most optimal care. On the basis of a deep learning approach, we performed unsupervised patient stratification of 19,636 patients with depression [major depressive disorder (MDD)] and/or schizophrenia (SCZ) and 22,467 population controls from the iPSYCH2012 case cohort. We integrated data of disorder severity, history of mental disorders and disease comorbidities, genetics, and medical birth data. From this, we stratified the individuals in six and seven unique clusters for MDD and SCZ, respectively. When censoring data until diagnosis, we could predict MDD clusters with areas under the curve (AUCs) of 0.54 to 0.80 and SCZ clusters with AUCs of 0.71 to 0.86. Overall cases and controls could be predicted with an AUC of 0.81, illustrating the utility of data-driven subgrouping in psychiatry.

## Teaser

Deep learning for data integration and stratification of schizophrenia and depression results in predictable subgroups.

## INTRODUCTION

The current psychiatric diagnostic categories remain restricted to subjective symptoms and subjectively observable signs, in contrast to most other medical fields, where diagnoses are often made based on quantifiable biomarkers. Schizophrenia (SCZ) and major depressive disorder (MDD) are both severe mental disorders with a large impact on the individual’s well-being and are among the biggest societal health burdens (*1*, *2*). Within the current diagnostic scheme of both SCZ and MDD, large variation exists in terms of treatment response, clinical presentation at onset, and disease progression (*3*). Furthermore, both SCZ and MDD have been shown to have polygenic architectures, associations with birth-related factors, as well as complex overlaps with other mental disorder diagnoses and general medical conditions, particularly diseases with immunological pathophysiological mechanisms, which could be used for stratification and prediction models (*4*–*6*). However, most prior studies have focused on the overall diagnostic categories and included a limited number of features, which might not be adequate to add the desired additional clinical value for these highly diverse disorders (*6*, *7*). Together, this calls for new and improved diagnoses and evaluations in psychiatry that can help stratify patients more efficiently and guide clinicians in providing optimal care (*6*).

For stratification of MDD and SCZ, a limited number of studies have applied machine learning for clustering, and no prior studies have used a deep learning (DL) framework (*8*–*10*). DL methods are able to process high-dimensional data and capture nonlinear structures (*6*, *11*). Specifically, variational autoencoders (VAEs), which are based on deep neural networks (NNs), have been highly useful for unsupervised learning of structures in large datasets (*12*). VAEs work by compressing high-dimensional data into lower-dimensional latent representations through the training of NNs, thereby capturing nonlinear correlations in the data. DL has shown promising results in identifying biologically relevant low-dimensional information from highly heterogeneous data such as transcriptomics, single-cell sequencing data, and integration of multiple datasets on human microbiome data (*13*–*16*). Currently, DL models have mainly been used to predict SCZ or MDD from data such as brain imaging via magnetic resonance imaging (MRI), genotype data, electroencephalographic, or social media data (*7*, *17*–*21*). However, these studies generally lack sufficient sample sizes (majority with no more than 400 cases with up to ~5500 for genetic models), have mainly looked at one diagnostic group, and lack true population controls to conclude on the generalization of the predictive power. To fully use the potential of the DL approach in gaining a deeper understanding of the complexity of the mental disorders, integration of more data from larger samples sizes is needed.

In this study, we used a large Danish population cohort from the Integrative Psychiatric Research Consortium (iPSYCH) of 19,636 individuals with MDD and/or SCZ including a population control group of 22,467 individuals, all genotyped and linked with the Danish nationwide registers (table S1). DL models were applied for efficient data integration to establish a clinically predictable stratification of individuals with MDD and SCZ. Individuals were stratified by all available data pertaining to them and their family medical history of mental disorders and other medical conditions, as well as birth-related variables, previously identified genetic markers from the literature, and the severity of their mental disorders presented by hospital contacts. Using the integrated data, we aimed to achieve a better stratification of possible disorder trajectories to get a more complete picture of the complexity between and within SCZ and MDD compared to a background population. Furthermore, by establishing new DL prediction models, we investigate the clinical predictability of the identified subgroups only including data up until their initial diagnosis. Given the size of the cohort and the fact that all individuals were followed from birth, we have increased power to gain new insights into the etiologies of these disorders using state-of-the-art DL methods.

## RESULTS

### Data-driven stratification of population controls and the severe mental disorders

**Fig. 1.**
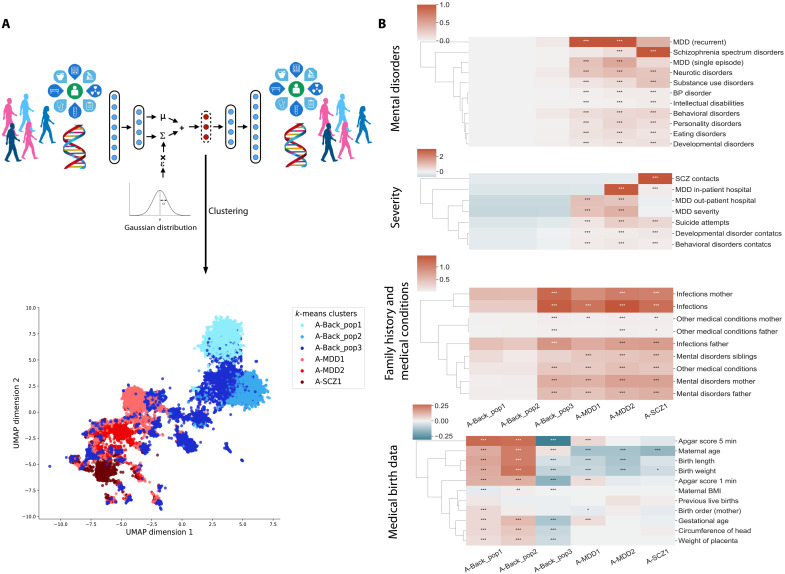
Clear stratification of depression and SCZ from background population subgroups using data-driven DL integration and clustering. (**A**) Overview of the VAE framework for integrating genetic data with all the available register data including their own and family history of diagnostic data for both mental disorders and immune-related disease, as well as birth-related measurements, previously identified genetic markers, and the severity of the mental disorders presented by hospital contacts, suicide attempts, and housing days. The overview illustrates how the VAE is used to learn the lower-dimensional latent representation for clustering of the individuals into distinct subgroups. The latent representation of the individuals is additionally used for a two-dimensional UMAP visualization to illustrate the clusters. The UMAP visualization is of all the individuals and illustrates the six identified clusters of background population, depression (MDD), and SCZ. Each dot represents a patient, and for GDPR (General Data Protection Regulation) purposes, we have masked all single occurrences not in close proximity with other individuals to ensure privacy (less than 30 individuals were masked in total). (**B**) Heatmap visualization of the cluster signatures identified in cluster analysis A. Here, we have for mental disorders grouped into the ICD-10 block within the F chapter, except for mood disorders that are divided into single-episode MDD, recurrent or single-episode MDD, and BD. The scale illustrates the fraction of individuals with at least one diagnosis within the ICD-10 block for that cluster. Both severity and medical birth data are *z*-score–normalized showing from low to high values. For severity, hospital contacts are a combined average of both days admitted and number of admissions. Family history and medical conditions are a combined count of the average number of occurrences across all included diagnoses per individual in the cluster. The significance levels are defined as *p < 0.05, **p < 0.001, and ***p < 0.0001.

We applied our DL VAE framework to integrate all the register-based and genetic data available (see Materials and Methods and data file S1) into a common latent representation to be used in the cluster analysis. Before clustering, the optimal model hyperparameters were determined on a random subset of 6000 individuals from the full cohort divided into training (5000) and test (1000) datasets to make it feasible for an exhaustive grid search (see Materials and Methods and fig. S1). In the cluster analysis of the full cohort of all 42,103 individuals, referred to as cluster analysis A, we identified an optimum of six clusters with an expected clear overall separation with three clusters characterized as background population (A-Back_pop1, A-Back_pop2, and A-Back_pop3), two clusters as MDD (A-MDD1 and A-MDD2), and one cluster in which 99% were diagnosed with SCZ (A-SCZ1) (Fig. 1, A and B, and fig. S2). Compared to using a standard principal components analysis (PCA), a sparse PCA (SPCA), a truncated singular value decomposition, or a Uniform Manifold Approximation and Projection for Dimension Reduction (UMAP) for reduction, the VAE showed a better separation of population control from mental disorders (fig. S3 and table S2). In the feature importance analysis, we found that psychiatric disorders and the severity of these had the largest impact on the clustering measured in the change of adjusted Rand index between subgroups (0.76 and 0.80), followed by family history and genetics (0.50 and 0.58) (table S3).

### Disorder clusters resemble the overall diagnostic categories

When investigating the signatures of the clusters, we found that two of the background population clusters, A-Back_pop1 and A-Back_pop2, did not have any individuals diagnosed with MDD, SCZ, or other mental disorders [Fig. 1B and data file S2 for all *P* values and confidence intervals (CIs)]. However, A-Back_pop3 had a higher similarity to the three primary mental disorder clusters and included individuals with infections [adjusted *P* value of <0.001 − average 1.38 per individual (pi)] and family history of mental disorders and infections; however, the average number of mental disorders (0.4 pi) was far lower compared to the disease clusters (2.7 to 4.4 pi). Furthermore, this cluster had a higher average polygenic risk score (PRS) for both MDD and SCZ than the two other background clusters (data file S2). The two MDD clusters were mainly separated by severity with A-MDD2 having more inpatient hospital contacts, suicide attempts, infections, and a higher PRS for MDD compared to A-MDD1 (all adjusted *P* values of <0.001) (data file S2). This highlights that, in broad terms, the overall diagnostic categories more or less form as separate distinct subgroups when compared to a background population, with the only subdivision being severe or less severe MDD.

### Excluding population controls gave a more detailed stratification of depression

**Fig. 2.**
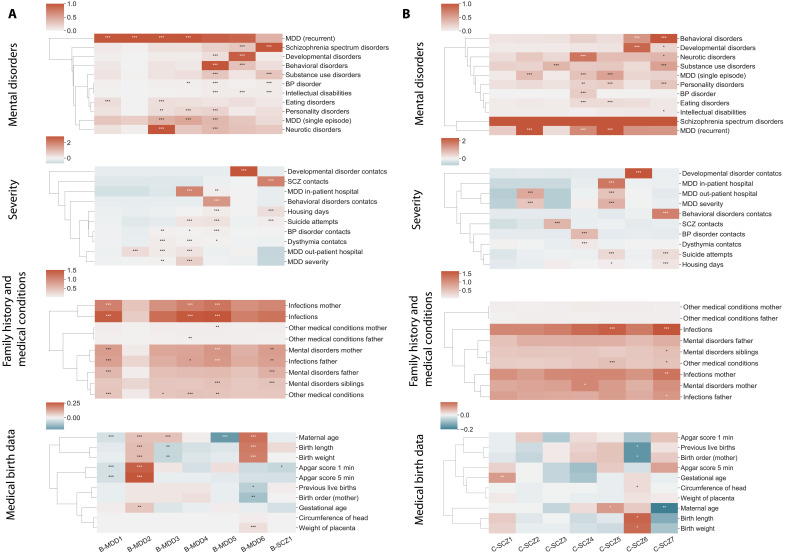
Distinct clinical signatures in both comorbidities and severity measurements for depression and SCZ subgroups. (**A**) Heatmap visualization of the cluster signatures identified in cluster analysis B of depression (MDD) and SCZ. Here, we have mental disorders grouped into the ICD-10 block within the F chapter, except for mood disorders that are divided into single-episode MDD, recurrent or single-episode MDD, and BD. The scale illustrates the fraction of individuals with at least one diagnosis within the ICD-10 block for that cluster. Both severity and medical birth data are *z*-score–normalized showing from low to high values. For severity, hospital contacts are a combined average of both days admitted and number of admissions. Family history and medical conditions are a combined count of the average number of occurrences across all included diagnoses per individual in the cluster. The significance levels are defined as *p < 0.05, **p < 0.001, and ***p < 0.0001. (**B**) Heatmap visualization of the cluster signatures identified in cluster analysis C of SCZ of the same data as described for the heatmaps in (A).

To get a deeper picture of possible subgroups within MDD and SCZ, we repeated the VAE integration and clustering of mental disorder cases only, referred to as cluster analysis B. Here, we identified seven clusters (fig. S4A), of which six were mainly MDD clusters (91 to 100%) and one resembled the A-SCZ1 cluster from cluster analysis A (B-SCZ1) (Fig. 2A and data file S3 for all *P* values and CI). Cluster B-MDD1 and B-MDD2 were the most similar of all clusters with a cluster distance of only 0.015 (fig. S5A) and were mainly distinguished by B-MDD1 having significantly more infections and other medical conditions and B-MDD2 having a slightly higher average of outpatient hospital contacts (Fig. 2A). Overall, the severity in terms of hospital contacts, suicide attempts, and mental comorbidities increased going from B-MDD1/B-MDD2 to B-MDD6 and to B-SCZ1. In cluster B-MDD3, 99.7% also had anxiety disorders; cluster B-BDD4 had more hospital contacts for MDD, suicide attempts, housing days, and infections; in B-MDD5, 99.9% had behavioral disorders with onset in childhood and adolescence; and in B-MDD6, 94.0% had developmental disorders. Furthermore, both B-MDD5 and B-SCZ1 were defined by more parental history of infections and mental disorders as well as more infections in B-MDD5. Both clusters were also highly severe in terms of suicide attempts and many inpatient hospital contacts for their MDD. Overall, the four clusters (B-MDD3, B-MDD5, B-MDD6, and B-SCZ1) with one other major mental comorbidity (>90%) had more additional mental comorbidities compared to the three other clusters (average of 4 to 6 pi compared to 1 to 2 pi).

For all clusters, we only observed some single-nucleotide polymorphisms (SNPs) with differences in homozygote/heterozygote distributions but not a single SNP or HLA signal that characterized the clusters (data file S3). In the SNP distribution and MDD-PRS, B-MDD2 was the most genetically different to the other subgroups and had a lower genetic load (PRS 3.9 × 10^−5^, *P* value of 0.0014), whereas A-MDD5 and A-MDD6 had the highest genetic load (PRS 4.1 × 10^−5^, *P* value of 0.0019 and 0.11). When looking at specific human leukocyte antigen (HLA) alleles that were at least nominally significantly associated with SCZ or MDD in previous studies (*22*, *23*), we observed that HLA*A0101 was significant in B-MDD2. Together, we identified clear subgroups within MDD with distinct disorder prognostics and comorbid signatures that highlight the high heterogeneity and potential clinical use to move toward a more personalized treatment strategy.

### Clear stratification of SCZ subgroups in terms of severity and comorbidities

For a more detailed stratification of the 3896 patients with SCZ, we conducted a subsequent analysis only including the patients diagnosed with SCZ referred to as cluster analysis C. We identified seven stable SCZ clusters from the latent representation with unique signatures across the included datasets (Fig. 2B and fig. S6A). Here, the feature importance showed a higher impact on each data modality, measured by the average change in the identified clusters using adjusted Rand index, compared to analyses A and B (0.47 compared to 0.44 and 0.38) (table S3). Of these, 62% had been diagnosed with MDD as well. In the two clusters, C-SCZ2 and C-SCZ5, all individuals were also diagnosed with MDD (single episode or recurrent) with C-SCZ5 having more severe MDD in terms of more inpatient and outpatient hospital contacts as well as more housing days and suicide attempts. C-SCZ5 had, in addition to the MDD diagnosis, a significantly higher average number of infections and other immune-related diseases per individual.

In general, we observed an increase in disorder severity with significantly more comorbidities, hospital contacts, suicide attempts, and housing days going from C-SCZ1 to C-SCZ7 (all adjusted *P* values <0.05; Fig. 2B and data file S4 for all *P* values and CI). The least severe cluster, C-SCZ1, had no significant fraction of other mental comorbidities and was defined by no significant increase in any severity measurements. C-SCZ3 was defined by having the most hospital contacts for their SCZ spectrum disorder and a higher amount of comorbid substance use disorder (21%). C-SCZ4 was characterized by a high fraction of multiple different mental disorder comorbidities (average of six mental disorder diagnosis pi) and a higher fraction of a maternal family history of mental disorders. We found that C-SCZ6 had a similar characteristic with B-MDD6 by having developmental disorders (89%) and an early average age of diagnosis for most of the comorbid mental disorders as well an early SCZ diagnosis of approximately 18 years (figs. S4B and S6B). The most severe cluster, C-SCZ7, had the highest average of housing days and suicide attempts as well as many comorbidities, within both mental disorders and infections. In this analysis, we found no significant signal for individual SNPs in the genetics data and only a few SNPs with significantly different homozygote/heterozygote distributions (data file S4).

### Prediction of cluster membership and MDD or SCZ diagnostics

To get an estimate of the clinical predictability of the clusters, we trained three feed-forward neural network (FFNN) prediction models for predictions of cluster membership on data up until their main mental disorder diagnosis of MDD or SCZ (prediction models A to C). To mimic a clinical situation at the time of diagnosis, we removed all disease severity measurements as well as events after the diagnosis of either MDD or SCZ including the diagnosis itself from the data used as input to the VAE. The time masked data were then used directly as input to the FFNN model for evaluating the prediagnostic predictability of clusters identified on the full-disorder trajectory. Other previous mental diagnoses were therefore still included; however, a diagnosis with MDD was only included in the prediction of the clusters in analysis C, to avoid introducing a biased increase in prediction accuracy for the patients with SCZ because of the nature of the cohort and the study setup. In addition, individuals without genotype data were removed from the prediction analysis to not bias the prediction due to imbalanced distribution of missingness between cases and controls. Furthermore, we, for comparison, trained a prediction model to predict the overall diagnostics groups of background, MDD, or SCZ using the same data (overall prediction model). All performance evaluations were done on a test set excluded from training and hyperparameter optimization (see Materials and Methods).

**Fig. 3.**
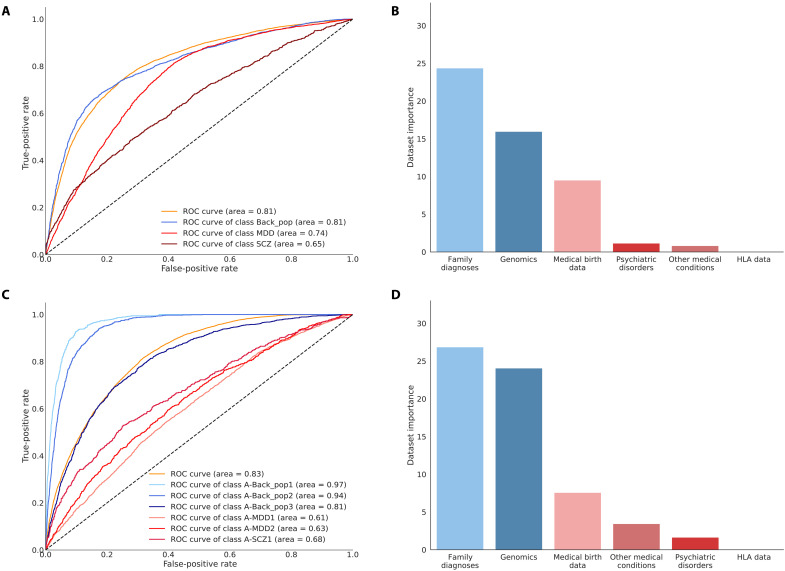
High predictability of subgroups and the broad diagnoses of depression and SCZ from a background population with clear impact from family history and genetics. (**A**) ROC-AUC or AUC for the performance of the prediction model and specifically for each of the diagnostic categories of control, depression (MDD), and SCZ. The model performance was calculated on the basis of the prediction on the randomly sampled test set. (**B**) The reduction in prediction accuracy across all categories in the prediction model when removing each of the datasets completely from the prediction on the test set. The dataset was removed by setting all features to the mean for continuous data (zero as the data are *z*-score–normalized) and to missing for categorical features (a zero vector in the one-hot encoding) and passing the test data through the pretrained prediction model. (**C**) ROC curves for prediction model A of the six clusters identified in cluster analysis A of all individuals with MDD, SCZ, and the background population, again showing the overall model performance across all clusters and the individual cluster performances. (**D**) The dataset-specific accuracy reduction for prediction model A calculated the same way as in (B). The colors are linked to the dataset.

The first overall prediction model of the current broad clinical diagnosis of MDD and SCZ showed good performance with an overall AUC of 0.81 (AUC 0.74 for MDD and AUC 0.65 for SCZ), a Matthew correlation coefficient (MCC) of 0.39, and an accuracy of 65.0% (multiclass by-chance accuracy 41.6%) on the test set (Fig. 3A and table S4). We identified misclassifications between all three categories suggesting similarities in the groups when only considering data before a possible diagnosis of MDD or SCZ (fig. S8). In prediction model A (Fig. 3C and table S4), we observed a comparable performance with an AUC of 0.83, an MCC of 0.37, and an accuracy of 49.1% (multiclass by-chance accuracy 21.3%). The highest individual cluster AUCs were for the background population clusters with AUCs of 0.81 to 0.97 most likely caused by the postdisorder data censoring not changing their input data. However, the results further support A-Back_pop3 (AUC of 0.81) being a high-risk group as most of the misclassifications between the mental disorder clusters and background population were predicted to be A-Back_pop3 (fig. S2C).

### Differences in the predictability of MDD and SCZ subgroups before diagnosis

**Fig. 4.**
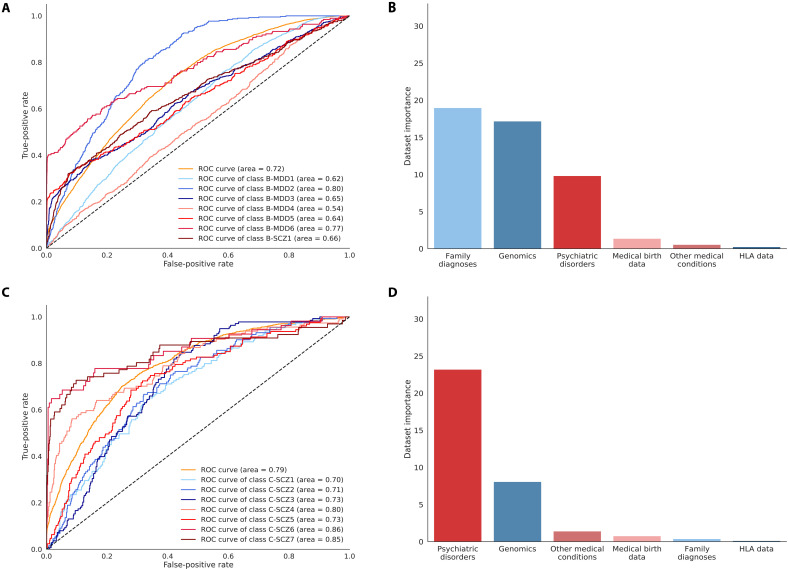
Subgroups within SCZ are easier to predict than within depression when only considering data before diagnosis. (**A**) ROC-AUC or AUC for the performance of the prediction model of the clusters identified in cluster analysis B of depression (MDD) and SCZ and the performance for each of the seven clusters. The model performance was calculated on the basis of the prediction on the randomly sampled test set. (**B**) The reduction in prediction accuracy across all categories in the prediction model when removing each of the datasets completely from the prediction on the test set. The dataset was removed by setting all features to the mean for continuous data (zero as the data are *z*-score–normalized) and to missing for categorical features (a zero vector in the one-hot encoding) and passing the test data through the pretrained prediction model. (**C**) ROC curves for prediction model C of the seven clusters identified in cluster analysis C of all individuals with MDD, SCZ, and the background population, again showing the overall model performance across all clusters and the individual cluster performances. (**D**) The dataset-specific accuracy reduction for prediction model B calculated the same way as in (B). The colors are linked to the dataset.

The SCZ subgroups in cluster analysis C were easier to predict with an AUC of 0.79, an MCC 0.29, and 40.5% accuracy (cluster AUCs ranging from 0.70 for C-SCZ1 to 0.86 for C-SCZ6) than the MDD subgroups in cluster analysis B with an AUC of 0.72, an MCC of 0.17, and 31.4% accuracy (cluster AUCs ranging from 0.54 for B-MDD4 to 0.80 for B-MDD2) (Fig. 4, A and C, and table S4). In general, we found that most clusters in both analyses B and C were highly similar in their data up until diagnosis, but specifically for cluster analysis B, some clusters were more or less identical, making prediction a complicated task (figs. S5 and S7). With the decrease in overall model performance, these results highlight that, in particular, individuals diagnosed with MDD were more homogeneous at the time of diagnosis, making the prediction of subgroup trajectories more difficult compared to the easier prediction task of diseased versus healthy.

### Feature importance for the prediction models

In both prediction models, which included the control population, the family history of diagnoses and genetics had the highest impact on the model accuracy; when removed, we observe a reduction in accuracy of 24.4 and 26.9% for the overall prediction model and prediction model A, respectively. Similarly, both models had genomics as the second most important feature with a reduction of 16.0 and 24.1%, followed by medical birth data with 9.5 and 7.6% (Fig. 3, B and D, and table S5). This pattern was also observed for prediction model B, with a 19% reduction for family history and a 17.2% reduction for genotype data. However, the patient’s own prior diagnoses of mental disorders and other medical comorbidities proved to be the third most important feature, with a 9.8% reduction (Fig. 4B and table S5). For prediction model C, we found that, in contrast to the other prediction models, the family diagnoses were among the least important (only 0.38% reduction), whereas the most important was the mental disorder diagnoses with a 23.2% reduction, followed by the genotype data with an 8.1% reduction (Fig. 4D and table S5). This shows that the prediction of SCZ subgroups is mainly based on earlier mental diagnoses. The general increase in the importance of each dataset in the prediction of the more detailed stratification suggests, as expected, that the clusters have a more complex structure that requires more variables to predict and thus removing a dataset causes a larger decrease in predictive power.

## DISCUSSION

In this first large-scale case-cohort study of 19,636 individuals with severe mental disorders and 22,467 population controls using DL for integration of genetics and registry data, we identified distinct subgroups with unique disorder severities and comorbidity signatures. Furthermore, we showed different predictability of the subgroups when only using data up until diagnosis ranging from AUC 0.55 to 0.97. Our DL data-driven stratification analysis clearly demonstrates the heterogeneity and overlap within and between mental disorder diagnostic categories of MDD and SCZ. Furthermore, several subgroups show high clinical predictability with an AUC above 0.8 for B-MDD2, C-SCZ4, C-SCZ6, and C-SCZ7. The important features defining our subgroups and used to predict them span both the pragmatic (based on disease-relevant features) and biological (based on causal factors) classifications, as described in the disease subtype framework recently proposed by Dahl and Zaitlen (*24*). This emphasizes the importance of our approach in the translational setting.

In previous studies, the predictive power of the model for a diagnosis of MDD and SCZ ranges between 0.54 and 0.95 in AUC or 45 to 99% accuracy (*7*, *17*, *19*). However, it was common for most models to have a questionable model evaluation and selection biases (*7*) and a low sample size of approximately 10 to 400 cases (up to ~5500 for genetic models) compared to ~4000 and ~16,000 cases of SCZ and MDD in our study, respectively. Furthermore, a direct comparison of the performances is not possible because our models are cross-diagnostic and the data types used in the studies are mostly different to ours, i.e., MRI and EGG (Electrogastrogram) data or models only relying on genetic data. In a real clinical setting, models that only predict one diagnosis compared to healthy individuals are not as clinically useful and would be comparable to our distinction of cases from population controls with an AUC of 0.81. However, our model has the improvement of a realistic control group as this also includes individuals with mental disorders, other than MDD and SCZ, thus being closer to a real clinical setting. As we also show here, cross-diagnostic signals exist between the mental disorders and a true population control group, which our prediction model encompasses. Therefore, there is a need for a better understanding of the cross-diagnosis similarities and differences to build a model that resembles a real clinical setting as many patients will likely present a continuum of symptoms within multiple mental disorders.

We identified an overlap in the characteristics of the subgroups identified within MDD to those within SCZ. For instance, we identified that both the MDD and SCZ cluster with high fraction of behavioral disorders, where B-MDD5 and C-SCZ7 had significantly more infections and other medical conditions compared to most other clusters as well as a lower maternal age. Similarly, we observed a high similarity between the two clusters with developmental disorders, B-MDD6 and C-SCZ6, with an earlier age at diagnosis across all comorbidities and a high predictability before diagnosis compared to the other clusters (AUC 0.77 and 0.86, respectively). This suggests that the subgroups of individuals where the majority is diagnosed with developmental disorders, mainly autism, tend to get their MDD or SCZ diagnosis earlier compared to the other subgroups. Consequently, the majority of the individuals in these clusters had a diagnosis that was given before MDD or SCZ diagnosis, which could improve the prediction.

For the prediction of clusters based on prediagnostic data, we do note that this will include partially overlapping data used for both clustering and prediction. While this is similar to a clinical application of such a prediction model of full-disorder trajectories, in a strict machine learning sense, this can result in an overestimation of the predictive performance. This potential effect on the performance measures is likely minor as most of the clusters were strongly driven by postdiagnostic features, such as hospital contacts, that were not included in the prediction models. Nonetheless, this should be considered when interpreting our performances compared with studies not using prediction for prediagnostic subgroup evaluation.

In all three cluster analyses, we did not find individual SNPs that were significantly overrepresented in the mental disorder subgroups or an overall separation in the genetic load of all SNPs. However, we did identify some significant differences in the distributions of the number of homozygote and heterozygote alleles for each SNP. Furthermore, the SNP distributions had predictive power in all the prediction models. This could be due to the fact that the presence or absence of a single SNP is not a strong enough signal to define the path of an entire subpopulation, and the underlying genetic signal is more likely to be a more complex combination of different genetic signatures (*25*, *26*). These were likely captured by the prediction model and resulted in the observed large impact of genomics data on the predictive power. This highlights the importance of the underlying genetics and their role in the prognostics of mental disorders. Overall, the signal from all combined HLA alleles was not strong.

One of the strengths of this study is the size of the cohort and the fact that it is a nationwide population-based cohort, which eliminates the majority of selection biases. However, the MDD cases are defined by having at least one visit to a hospital and, thus, only include the most severe MDD cases. Those with a less severe depression diagnosis will only have visits to general practitioners (GPs) and will not be registered in the hospital-based registers. This might explain some of the homogeneity we observe in the data before diagnosis and why many of these individuals might resemble the individuals diagnosed with SCZ. It is also likely that the background population individuals in the high-risk A-Back_pop3 cluster have been diagnosed with depression by their GP without being severely depressed, at least not within the time constrained of the study. From the data available in this analysis, we also have only a snapshot of the diagnosis data and an age of the first diagnosis. Some important differences might be observed in the number of repeated infections or severity of the infections for which data were not available to us.

Using DL approaches, we identified clinically distinct subgroups within both MDD and SCZ underlining the potential added values of a deeper stratification rather than only using the broad diagnostic categories. With this study being conducted on one of the largest cohorts in the field, we have shown that we can predict the most likely subgroup of either background population or MDD and SCZ as well as predict the specific subgroup within MDD and SCZ. With more detailed clinical information, such as omics data, EGG and MRI data, detailed clinical health records, and clinical assessments data with more information on treatment responses from clinical cohorts, the potential response differences within the subgroups can be further addressed. Thus, integrating the models presented in this study with further large-scale studies with more detailed molecular profiles and psychopathology would allow us to investigate the exact mechanisms involved and gain further insights into this proof-of-concept study. With further insights into the subgroups, these clinical prediction models can assist clinicians in the diagnosis and prognostics of patients with mental disorders providing more standardized and personalized care to the benefit of the individual patient.

## MATERIALS AND METHODS

### Cohort and experimental setup

The data in this study are from the large Danish population-based case-cohort sample (iPSYCH2012) from the iPSYCH (*27*). The iPSYCH2012 case-cohort sample consists of 87,764 Danish individuals who were born in Denmark between 1981 and 2005 and had dry blood spots from samples taken at birth available from the Danish Neonatal Screening Biobank (*28*). In this cohort, there are 57,764 people with at least one major mental disorder and 30,000 individuals who were randomly sampled from the population without regard for the psychiatric disorders. The mental disorders included in the iPSYCH cohort are autism spectrum disorder (F84.0-1, F84.5, and F84.8-9), attention-deficit/hyperactivity disorder (ADHD) (F90.0), SCZ (F20), bipolar disorder (BD) (F31), and MDD (F32 and F33). Genotyping, data cleaning, and imputation were described in detail by Schork *et al.* (*29*). For this study, we only included the individuals diagnosed with SCZ and/or MDD based on the International Classification of Diseases (ICD) 10 F20 and F32-F33 [ICD-8 codes 295.x9 (excl 295.79) for SCZ and 296.09, 296.29, 298.09, and 300.49 for MDD]. This included 19,636 individuals with either MDD or SCZ, of whom 15,740 were diagnosed with MDD and 3896 were diagnosed with SCZ. Furthermore, a population control group of approximately the same size, 22,467, was randomly selected from the control population in the iPSYCH cohort, not including individuals with either of the two focus diagnoses. From the Danish Psychiatric Central Research Register (*30*), we included all psychiatric diagnoses in ICD-10/ICD-8 codes including information on the age of diagnosis and psychiatric-related hospital contacts. In addition, registered infections, autoimmune diseases, the medical birth registry including complications and birth weight, and a selected number of other diseases were included. For the family history of the included individual, we only included parental psychiatric diagnoses, and siblings were included as a combined count of how many with the specific diagnoses. All data from the registers were included up until 2016 for mental disorders and 2012 for infections and autoimmune diseases. A total of 1185 variables were included before preprocessing of the data.

### Preprocessing of data

The genotype data were included as the genotypes of risk alleles toward MDD, SCZ, bipolar spectrum disorder (BD), autism, ADHD, suicide ideation, autoimmune diseases, or infections such as influenza, human immunodeficiency virus, and hepatitis. The alleles were identified from the genome-wide association study (GWAS) catalog (*31*) and only included if the risk allele was specified. Note that, with regard to the genetic data, individuals who failed the genetic quality control as described in Schork *et al.* (*29*) were treated as having missing data. The quality control included removal of individuals with non-European descent as well as related individuals. We additionally ran a PCA using SmartPCA (*32*) on the 31,863 individuals with genotype data together with part of the HapMap sample [Japanese (JPT), Chinese (CHB), Yoruba (YRI), and European (CEU) descent] (*33*) to test for any major ancestral structure among the individuals in the 516 included SNPs. The cohort clustered with the European individuals, as expected, and showed no clear indication of ancestral structures (fig. S9). Furthermore, we found that none of the 10 first PCs were strongly correlated with the clusters (all PCC < 0.1). The genotype and HLA alleles were both included as being either homozygote for the allele, heterozygote, or not having the allele. The continuous data variables such as age of diagnosis, the number of hospitalizations, patient contacts, and birth measures were all normalized and centered around zero by *z*-score normalization per feature. Missing continuous data were encoded as the mean, which, for z-score–normalized data, is zero. The categorical data, genotype, HLA, and family history, were one-hot–encoded and flattened for input to the model. All missing categorical data were encoded as a zero vector. We only included data that were observed in at least 1% of the individuals in the analysis, and therefore, we had a small difference in the number of features including analyses A to C with 668, 692, and 711 features in A to C, respectively (see data file S1 for the full list). For the prediction models, we applied an individual-based age masking of the input data to resemble a clinical setting at the time of the diagnosis with either MDD or SCZ. This included masking of all diagnosis given after the exact age of MDD and SCZ including the diagnosis itself. Therefore, individuals with previous mental disorder diagnoses would still have these available for the prediction. Previous diagnoses of MDD were excluded from all prediction models that included both patients with MDD and SCZ as this would create a bias because only patients with SCZ could have this diagnosis after the masking. All severity measurements (hospital contacts and suicide attempts) were removed as these were available as a total sum of contacts and not individual events.

### DL model

The DL model used to integrate the data was a VAE (*12*). A VAE consists of an encoder network, followed by a latent layer of size NL that is passed on to a decoder of the same sizes as the encoder layers arranged in reversed order. The model framework was built to account for a variable number of fully connected hidden layers in both the encoder and decoder. Each hidden layer included both batch normalization (*34*) and dropout (*P* = 0.1) (*35*) with Leaky Rectified Linear Units (*36*) as activation function. The latent layer was built from sampling from a Gaussian distribution N(0,I) of two fully connected layers of the means (μ) and SDs (σ), both of size NL.

When training the model, each dataset was merged to one input layer including both categorical and continuous variables and passed through the network. The reconstruction error was calculated separately for categorical and continuous datasets by splitting up the reconstructed output vector. The loss functions applied were cross-entropy for categorical data and mean-squared error for continuous data as implemented in PyTorch. For the categorical variables, we avoided back propagating of missing values using the ignore index implementation in PyTorch. For continuous variables, we set the reconstructions for all missing values to zero to match the input, so these would not contribute to the loss. In addition, the sampling of the latent layer was constrained to the Gaussian distribution by penalizing the deviance by adding the Kullback-Leibler divergence (KLD) to the loss. The final loss was defined as

$$L=E_{\text{cat}}+E_{\text{con}}+W_{\text{KLD}}\times \text{KLD}$$

Here, *E*_cat_ and *E*_con_ are the normalized reconstruction error for the continuous and categorical data. *W*_KLD_ is a weight put on the KLD defined as *W*_KLD_ = β × *N_L_*^−1^, for which we used a β of 0.0001 for the final model. The KLD was defined as

$$\text{KLD}=-\frac{1}{2}\times \sum 1+\text{ln}(\sigma )-\mu -\sigma$$

The VAE model was trained with the Adam optimizer (*37*) and used the same settings to train three models for each analysis referred to as models A, B, and C. Between the models, the only changes in the training parameters were the batch size due to the differences in the sizes of the subsets of the data. Here, we used a batch size of 30, 15, and 10 for models A to C, respectively. We increased the batch size by a factor of 1.5 during training after every 50 epochs and used KLD warm-up by slowly increasing WKLD from zero to β × NL-1 at epochs 4, 6, 8, and 10 (*38*). The number of training epochs was determined to be 250 based on early stopping on the test set described under the hyperparameter testing. Hereafter, the latent representation of each individual was obtained by passing them through the trained VAE and extracting the μ layer. The VAE was implemented using PyTorch (v.1.4.0) (*39*).

### Hyperparameter selection and stability testing

To identify the optimal parameters in terms of reconstruction accuracy, cluster capability, and model stability, different combinations of size of hidden layers, number of hidden layers, and size of latent space were evaluated. For the number of hidden neurons, we tested the sizes 400, 600, and 800, with the number of layers ranging between 1 and 3 with the same number of hidden neurons in each layer. The number of latent neurons used was 10, 20, or 40. We did an exhaustive search of all combinations on a randomly selected sample of 5000 individuals from the whole dataset for training and another 1000 individuals as a test set. The model performance was then evaluated in terms of test log-likelihood and reconstruction accuracy as well as reconstruction accuracy on the training data. An accurate reconstruction was for categorical variables defined as the class with the highest probability corresponding to the class given by the input. For continuous variables, the accuracy was assessed by comparing the reconstructed array with the input array using cosine similarity for each feature. Only nonmissing values were used when calculating the accuracy in the reconstruction. The performance of the model was also assessed by comparing the ability to cluster the data in terms of the intercluster separation calculated as the sum of squared distances (see more detail under the “Clustering” section). We chose the number of training epochs based on the lowest test error during training and rounded up to the nearest 100 epochs to ensure sufficient training to learn the complexity of the data. The stability of the model was evaluated by repeating training three times with the same hyperparameters and calculating the difference in cosine similarity to all other individuals for each individual in the dataset. If the model produced the same result, the average change in cosine similarity should be zero. The model with the average change closest to zero is then considered the most stable. The final model was then selected on the basis of all the above performance measures by selecting the model with the highest total rank across all tests. From this analysis, the optimal network architecture was found to be two hidden layers of 800 neurons and a latent space size of 40 dimensions (fig. S1).

### Clustering

The clustering of the data was done using *k*-means, and all clustering and testing were done using Python scikit-learn (v.0.21.3) (*40*). The optimal number of clusters for the *k*-means approach was determined using the silhouette score to measure the spread of the clusters as well as the elbow method using the sum of squared distances to measure the intercluster separation as implemented in the scikit-learn package. From the latter, the optimal number of clusters was determined by first calculating the sum of squared distances (SSDs) of every number of clusters. From the SSD for the smallest and largest number of clusters, we then fitted a linear line and selected the optimal cluster number as the one with the largest distance to this line. The final clusters were selected on the basis of *k*-means clustering on a consensus matrix of repeated clustering of the latent space using the same parameters to ensure stable clusters.

### Comparing PCA and UMAP-based reduction for clustering

We tested whether performing the dimensionality reduction before the cluster analysis using other methods would give similar results. Here, we used a PCA, UMAP (*41*), SPCA, and truncated singular value decomposition (TSVD) with the same reduction size (40 dimensions) (fig. S3). Here, we identified some overlap in cluster signatures when identifying the same number of clusters with adjusted Rand indices of 0.35, 0.23, 0.40, and 0.42 for PCA, UMAP, SPCA, and TSVD, respectively, using Python SciPy (v.1.3.1) (*42*). However, these clusters had a worse separation of population control from mental disorders. Using PCA and UMAP, we only found one clear background population (98 to 100%) cluster and two mixed (17 to 85% in background population clusters for PCA and 18 to 90% for UMAP) (fig. S3 and table S2). When based on UMAP reduction, no clusters were identified without any background population (3 to 13% in the remaining three clusters). SPCA and TSVD were both closer to the VAE clustering in the distribution of diagnoses in the main disorder groups (two 100% MDD with between 6 and 8% or between 12 and 14% SCZ and one SCZ cluster with 44 to 47% MDD). However, the background clusters had a higher percent of the cases with only 83 to 86% as background. This highlights the added value of using the VAE for dataset reduction with the two sparse methods, SPCA and TSVD, showing better performance compared with PCA and UMAP.

### Assessment of impact of genotype missingness on clustering

The VAE integration can handle missing data by not using that information in the generation of the latent space. In our analysis, we had 10% of the individuals not having genotype data available because of failed QC (quality control). Of these, the majority was within the background population group, which had 18% missing data. We tested our VAE integration and cluster analysis without including these individuals and were able to identify the same six groups of three background groups, two MDD subgroups, and one SCZ subgroup (fig. S10). Therefore, we concluded that our method is stable and can handle a high percent missingness that is unevenly distributed between the labels included in the analysis.

### Feature importance on clustering

We calculated the impact of each data modality on the clustering by setting each of the datasets to missing before VAE integration. We then recalculated the clusters by using the same *k*-means approach. The impact was then calculated from the adjusted Rand index between the true labels and the labels identified with the dataset set to missing. The reported impact is the change from a perfect overlap of 1.

### Polygenic risk score calculation

PRSs for MDD and SCZ were calculated for iPSYCH individuals using PRSice2 v2.3.3 (*43*) and summary statistics from a meta-analysis of GWAS of MDD and SCZ. The summary statistics were based on Psychiatric Genomics Consortium samples from which Danish samples were excluded before meta-analysis. The MDD sample included 49,982 cases and 136,871 controls, and the SCZ sample included 34,129 cases and 45,512 controls (*44*, *45*). We included all SNPs in the calculation using a *P* value threshold of 1 and used a clumping r2 threshold of 0.1 in a window of 250 kilo–base pairs. The “--score” sum method was used to calculate the scores. Otherwise, the default parameters were used.

### Statistical analysis

The identification of cluster-defining variables was done using a chi-square test for categorical variables using Python SciPy (v.1.3.1) (*42*). For the mental diagnoses, calculation was done by combining all diagnoses within the same block in the ICD-10 system and ICD-8 codes included in the corresponding ICD-10 block, and calculating the fraction of the individuals in a given cluster with at least one diagnosis within each block in the chapter. For the other medical conditions, these were combined into infections and others (migraine, diabetes, etc.) by counting the average number of each type of the two conditions each individual had within each cluster. The same calculation was done for the family history separated both into mental diagnoses and other medical conditions and infections as well as parental and maternal. For siblings, only mental diagnosis for MDD, BPD, SCZ, or any was available and included as a fraction of individuals with siblings with a diagnosis within each cluster. Calculation of genetic signals for both disorder-related phenotype SNPs and HLA alleles was done as the number of alleles out of all possible alleles within each cluster. All statistical tests for these were calculated as categorical with a one-sided chi-square test for identification of overrepresentation and cluster defining of diagnoses for each cluster. Furthermore, we also calculated whether there was a difference in the distribution of being a homozygote and being a heterozygote for each SNP with a two-sided chi-square test. For the medical birth data and severity variables, the numbers were included as the *z*-score–normalized values. For the medical birth data, a two-sided Student’s *t* test was used to calculate whether a significant higher or lower, e.g., birth weight was defining the cluster. The hospital contacts for the severity measurements were combined as an average across all variables for the disorder (such as number of contacts and days admitted). For MDD, these were separated into outpatient and inpatient hospital contacts, and the statistics were done with a one-sided Student’s *t* test to test whether a higher severity was defining the cluster. All tests were corrected for multiple testing using Bonferroni correction, and all the *P* values reported are corrected.

### Cluster visualization and distances

We applied UMAP (*41*) on the latent representations for visualizing the clusters in two dimensions to capture potential nonlinear structures. The reduction was only applied for visualization purposes and not used to define the clusters or infer any relationships or distances between clusters. Cluster distances are computed as the correlation distance between the mean values of each cluster pair using the “distance.correlation” from the SciPy python package (v.1.3.1) (*42*). The distance between clusters ranges from 0 to 1, with 1 being the maximum distance.

### Prediction models

The prediction models for the subsequent prediction of cluster labels and main disorder group (MDD, SZC, or background population control) were performed using an FFNN implemented using Python PyTorch (v.1.4.0) (*39*). We initially also tested the performance using a random forest model, using scikit-learn (v.0.21.3) (*40*); however, it was unable to handle the class imbalance compared to the performance of the FFNN with overprediction of the largest class (MCC of 0.26 in analysis A). We trained the model on 80% of the data and used the remaining 20% as a test set to evaluate the model. In the training, we used 10% of the data as a validation set for the hyperparameter optimization. The data were split using the train_test_split function implemented in Python scikit-learn (v.0.21.3) (*40*) to ensure equal splits across classes. Furthermore, we used the WeightedRandomSampler in PyTorch to account for class imbalance. We used the same implementations of activation function, dropout, and batch normalization as in the VAE and cross-entropy for loss calculation. The hyperparameter optimization was done with a full grid search of all combinations, and the model with the lowest prediction error on the validation set was used to evaluate the model. Early stopping was applied by saving the model with the lowest validation error during the 200 epochs of model training. On the basis of the performance of the VAE, we only tested prediction models with two hidden layers with the sizes [64, 32], [128, 64], or [256, 128]. Furthermore, we tested the learning rate (0.001 or 0.0001) and the batch size with different ranges depending on the analysis ([40, 50, 100] for analysis A, [20, 30, 40, 50] for analysis B, and [5, 10, 20, 25] for analysis C). The best-performing model was evaluated on the test set by calculating the receiver operating characteristic area under the curve (ROC-AUC or AUC) of the predictive power of each of the clusters individually and across all clusters. Furthermore, we calculated the overall accuracy and the MCC for each model. All performance calculations were done using the scikit-learn implementations. For all randomizations, the seed was set to 42. In the prediction model, all individuals with missing genetic data were removed in analysis A due to the large bias in the distribution between background and cases that only our VAE integration and clustering could account for. This removed around 10% of the individuals from the model, leaving 31,863 individuals in total. For analysis A, the optimal hyperparameter for the best-performing model on the validation set was a batch size of 100, a learning rate of 0.0001, and two layers of 256 and 128 neurons. For the second model in analysis A on predicting MDD, SCZ, or background, we used the same hyperparameters identified for prediction of the cluster membership. For analysis B, we found the optimal hyperparameters to have a batch size of 50, a learning rate of 0.0001, and two layers of 256 and 128 neurons cluster membership, which were also used in the MDD or SCZ prediction. Last, the parameters for the prediction model in analysis C were a batch size of 10, a learning rate of 0.0001, and two layers of 256 and 128.

## Notes

View/request a protocol for this paper from *Bio-protocol*.
