# Supplementary material for: Deep learning–based integration of genetics with registry data for stratification of schizophrenia and depression
Source: Sci Adv. 2022 Jun 29;8(26):eabi7293. doi: 10.1126/sciadv.abi7293 (PMC9242585; doi:10.1126/sciadv.abi7293)
Supplement: Supplementary file 1 — Figs. S1 to S10 Tables S1 to S5 [file sciadv.abi7293_sm.pdf]

Science Advances
AAAS

Supplementary Materials for

Deep learning–based integration of genetics with registry data for stratification of schizophrenia and depression

Rosa Lundbye Allesøe *et al.*

Corresponding author: Simon Rasmussen, simon.rasmussen@cpr.ku.dk; Michael Eriksen Benros, michael.eriksen.benros@regionh.dk

Sci. Adv. **8**, eabi7293 (2022)
DOI: 10.1126/sciadv.abi7293

The PDF file includes:

Figs. S1 to S10
Tables S1 to S5
Legends for data S1 to S4

Other Supplementary Material for this manuscript includes the following:

Data S1 to S4

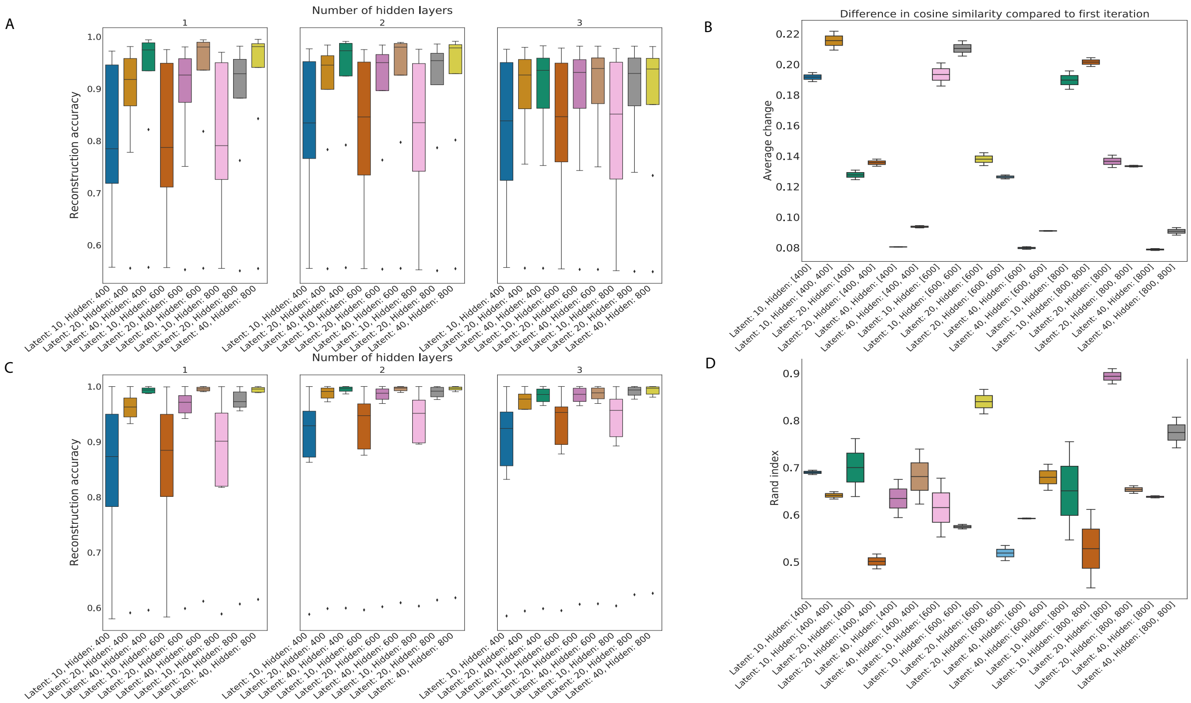

Fig. S1. Hyperparameter optimization results for training the Variational Autoencoder. **(A)** Distribution of reconstruction accuracies on the training data of each dataset for the tested hyperparameter combinations. **(B)** Stability of the latent space for different hyperparameters calculated as the average absolute change in cosine similarity between the latent representation of each individual in the first iteration of training and each of the five repeated trainings **(C)** Distribution of reconstruction accuracies on the test data withhold from training of each dataset for the tested hyperparameter combinations **(D)** Stability of the clustering calculated as the Rand index between repeating clustering of the latent space using different hyperparameter settings.

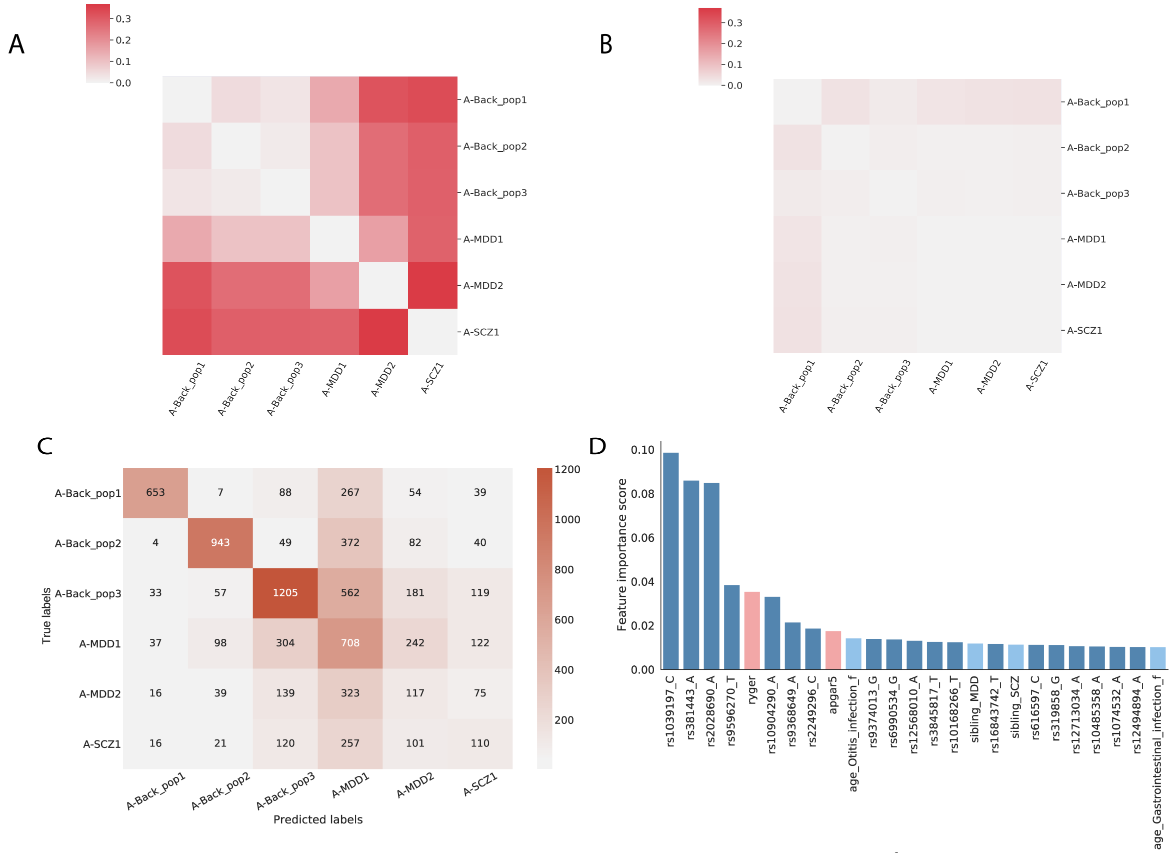

Fig. S2. Cluster similarity and prediction model performance for analysis A.

(A) Correlation distance between the 6 identified clusters in cluster analysis A including all individuals diagnosed with depression (MDD) and/or schizophrenia (SCZ) as well as the background population control group (Back_pop). The distances are calculated based on all included genetic and register data including their own and family history of diagnostic data for both mental disorders and immune related disease, as well as birth related measurements and the severity of the mental disorders presented by hospital contacts, suicide attempts and housing days. **(B)** Correlation distance between the clusters when only considering all events prior to diagnosis of MDD or SCZ. **(C)** Confusion matrix showing the distribution of true and false predictions for prediction model A for predicting the subgroups identified for MDD, SCZ and background population. **(D)** Single feature impact on prediction accuracy for the prediction model A of the clusters in cluster analysis A. Colors indicate type of dataset with dark blue being genetics, light blue family diagnosis and light red medical birth data.

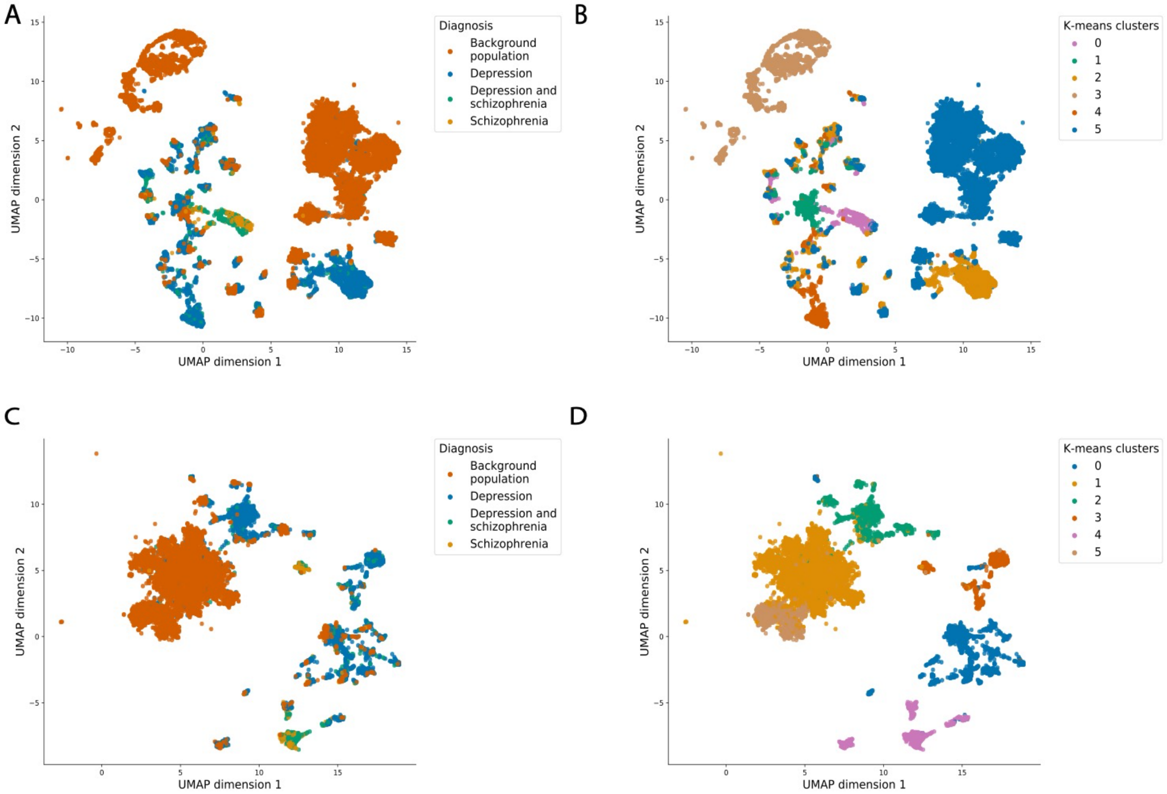

Fig. S3. Comparison to principal component analysis for dimensionality reduction.

(A) Two-dimensional UMAP visualization of the PCA reduction of the same data used as input to the VAE illustrating the overall broad diagnostic categories of depression, schizophrenia, both disorders or background population group. The UMAP visualization is of all the individuals where each dot represents a patient and for GDPR purposes we have masked all single occurrences not in close proximity with other individuals to ensure privacy (less than 30 individuals were masked in total). **(B)** The same two-dimensional UMAP visualization of the PCA as in (A) colored by the 6 clusters identified using k-means clustering. **(C)** Two-dimensional UMAP visualization of the UMAP reduction of the same data used as input to the VAE and PCA illustrating the overall broad diagnostic categories of depression, schizophrenia, both disorders or background population group. The UMAP visualization is of all the individuals where each dot represents a patient and for GDPR purposes we have masked all single occurrences not in close proximity with other individuals to ensure privacy (less than 30 individuals were masked in total). **(D)** The same two-dimensional UMAP visualization of the UMAP as in (C) colored by the 6 clusters identified using k-means clustering.

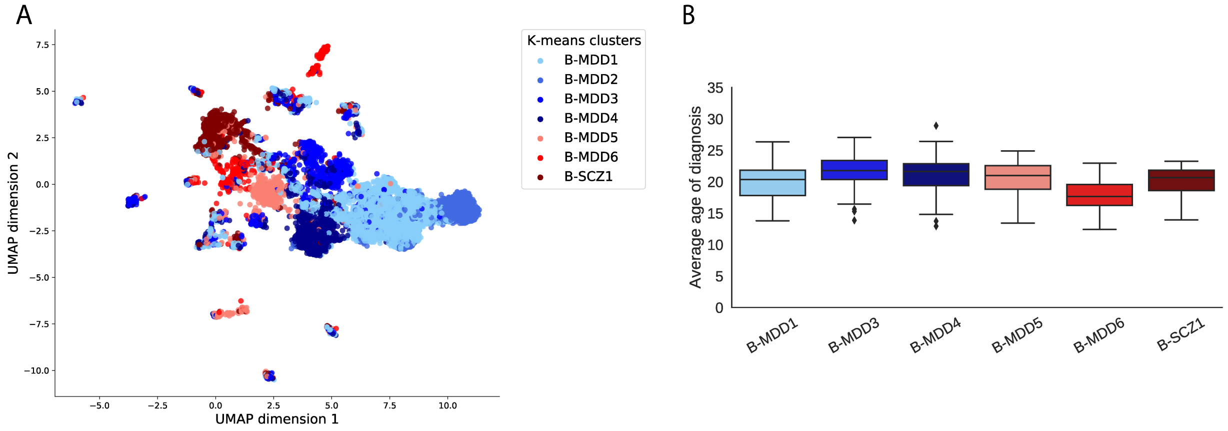

Fig. S4. Visualization and age distribution of disorder onset for cluster analysis B.

(A) Two-dimensional UMAP visualization of the latent representation of all individuals diagnosed with depression (MDD) and/or schizophrenia (SCZ) used in cluster analysis B. The UMAP visualization illustrates the 7 identified clusters of subgroups within MDD and one SCZ cluster. Each dot represents a patient and for GDPR purposes we have masked all single occurrences not in close proximity with other individuals to ensure privacy (less than 20 individuals were masked in total). **(B)** The distribution of the average age of mental disorder one-sets for all diagnosis within the ICD-10 F chapter included in the dataset across all the 7 identified clusters in cluster analysis B

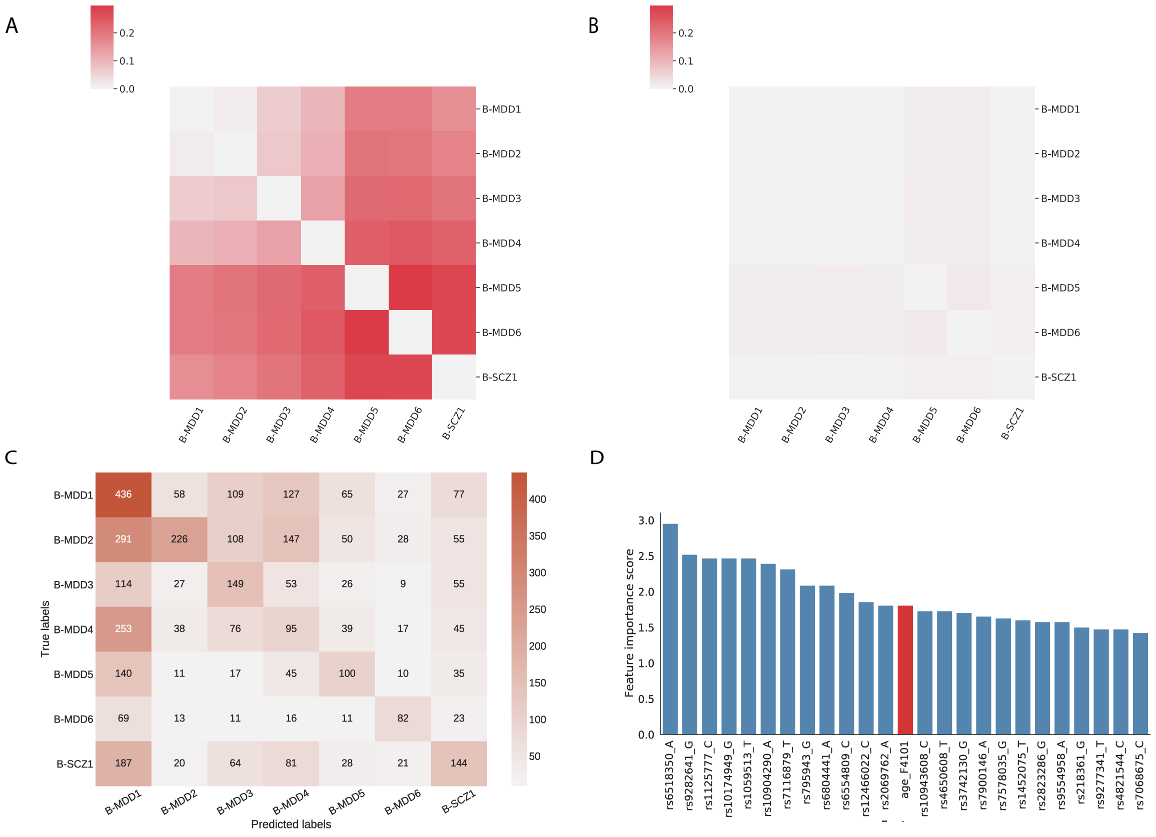

Fig. S5. Cluster similarity and prediction model performance for analysis B.

(A) Correlation distance between the 7 identified clusters in cluster analysis B including all individuals diagnosed with depression (MDD) and/or schizophrenia (SCZ). The distances are calculated based on all included genetic and register data including their own and family history of diagnostic data for both mental disorders and immune related disease, as well as birth related measurements and the severity of the mental disorders presented by hospital contacts, suicide attempts and housing days. **(B)** Correlation distance between the clusters when only considering all events prior to diagnosis of MDD or SCZ. **(C)** Confusion matrix showing the distribution of true and false predictions for prediction model B for predicting the subgroups identified for MDD and SCZ. **(D)** Single feature impact on prediction accuracy for the prediction model B of the clusters in cluster analysis B. Colors indicate type of dataset with dark blue being genetics and red being own diagnosis.

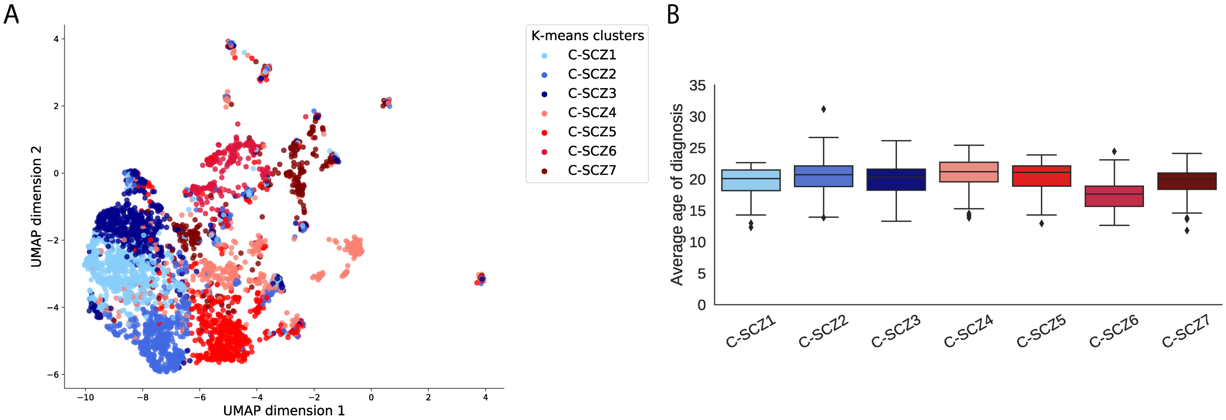

Fig. S6. Visualization and age distribution of disorder onset for cluster analysis C.

(A) Two-dimensional UMAP visualization of the latent representation of all individuals diagnosed with schizophrenia (SCZ) used in cluster analysis C. The UMAP visualization illustrates the 7 identified clusters of subgroups within SCZ. Each dot represents a patient and for GDPR purposes we have masked all single occurrences not in close proximity with other individuals to ensure privacy (less than 20 individuals were masked in total). **(B)** The distribution of the average age of mental disorder one-sets for all diagnosis within the ICD-10 F chapter included in the dataset across all the 7 identified clusters in cluster analysis C

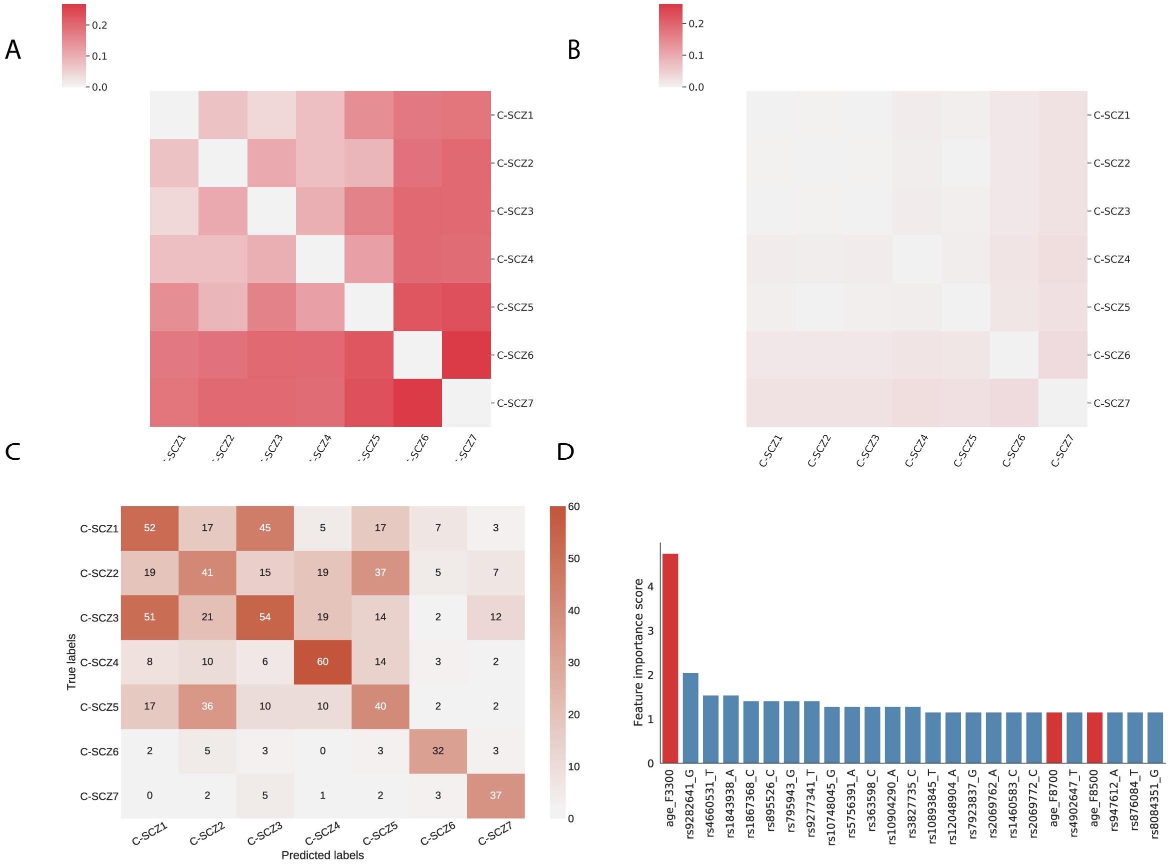

Fig. S7. Cluster similarity and prediction model performance for analysis C.

(A) Correlation distance between the 7 identified clusters in cluster analysis C including all individuals diagnosed with schizophrenia (SCZ). The distances are calculated based on all included genetic and register data including their own and family history of diagnostic data for both mental disorders and immune related disease, as well as birth related measurements and the severity of the mental disorders presented by hospital contacts, suicide attempts and housing days. **(B)** Correlation distance between the clusters when only considering all events prior to diagnosis of SCZ. **(C)** Confusion matrix showing the distribution of true and false predictions for prediction model C for predicting the subgroups identified for SCZ. **(D)** Single feature impact on prediction accuracy for the prediction model C of the clusters in cluster analysis C. Colors indicate type of dataset with dark blue being genetics and red being own diagnosis.

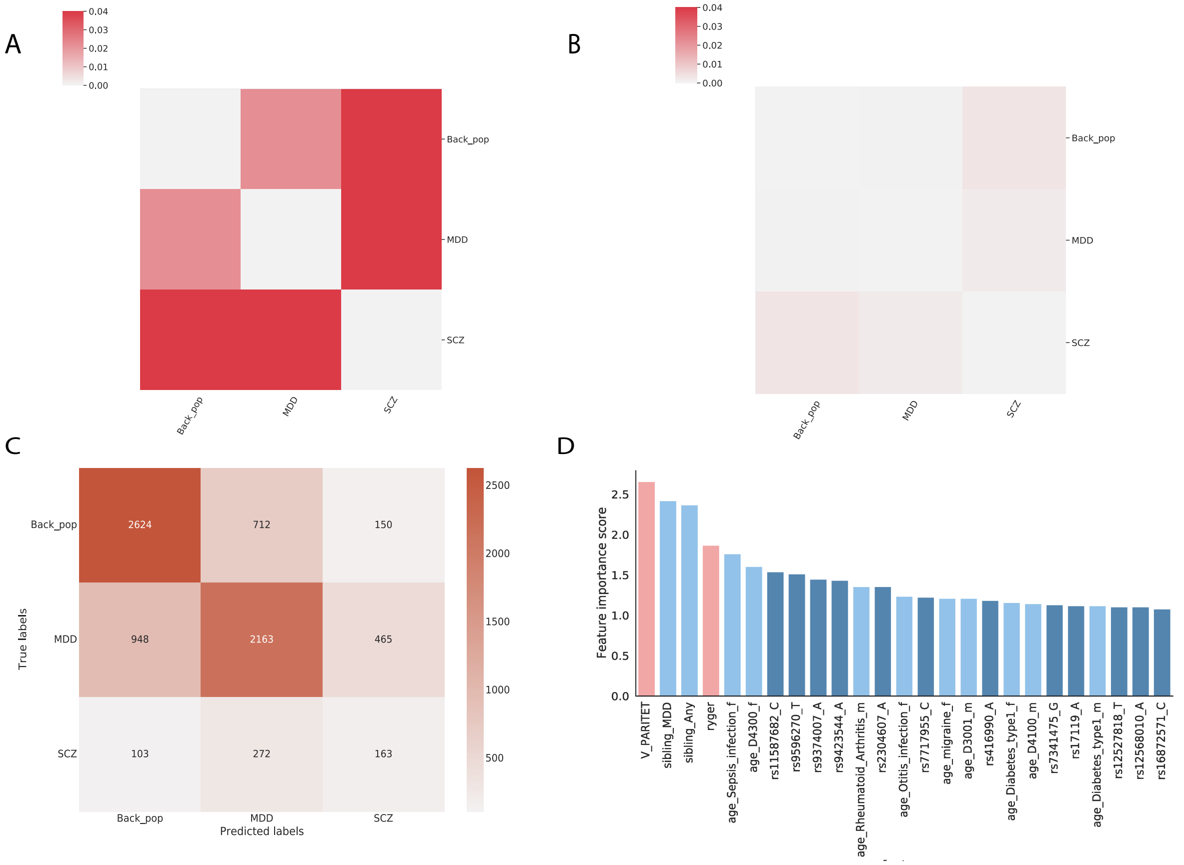

Fig. S8. Diagnostic group similarity and prediction model performance for overall prediction model.

(A) Correlation distance between the broad diagnostic categories of depression (MDD), schizophrenia (SCZ) and background population (Back_pop) across all included genetic and register data including their own and family history of diagnostic data for both mental disorders and immune related disease, as well as birth related measurements and the severity of the mental disorders presented by hospital contacts, suicide attempts and housing days. **(B)** Correlation distance between the broad diagnostic categories when only considering all events prior to diagnosis of MDD or SCZ. **(C)** Confusion matrix showing the distribution of true and false predictions for the prediction model for predicting the broad diagnostic categories of MDD, SCZ and background population. **(D)** Single feature impact on prediction accuracy for the prediction model of the broad diagnostic categories. Colors indicate type of dataset with dark blue being genetics, light blue family diagnosis, light red medical birth data and red being own diagnosis.

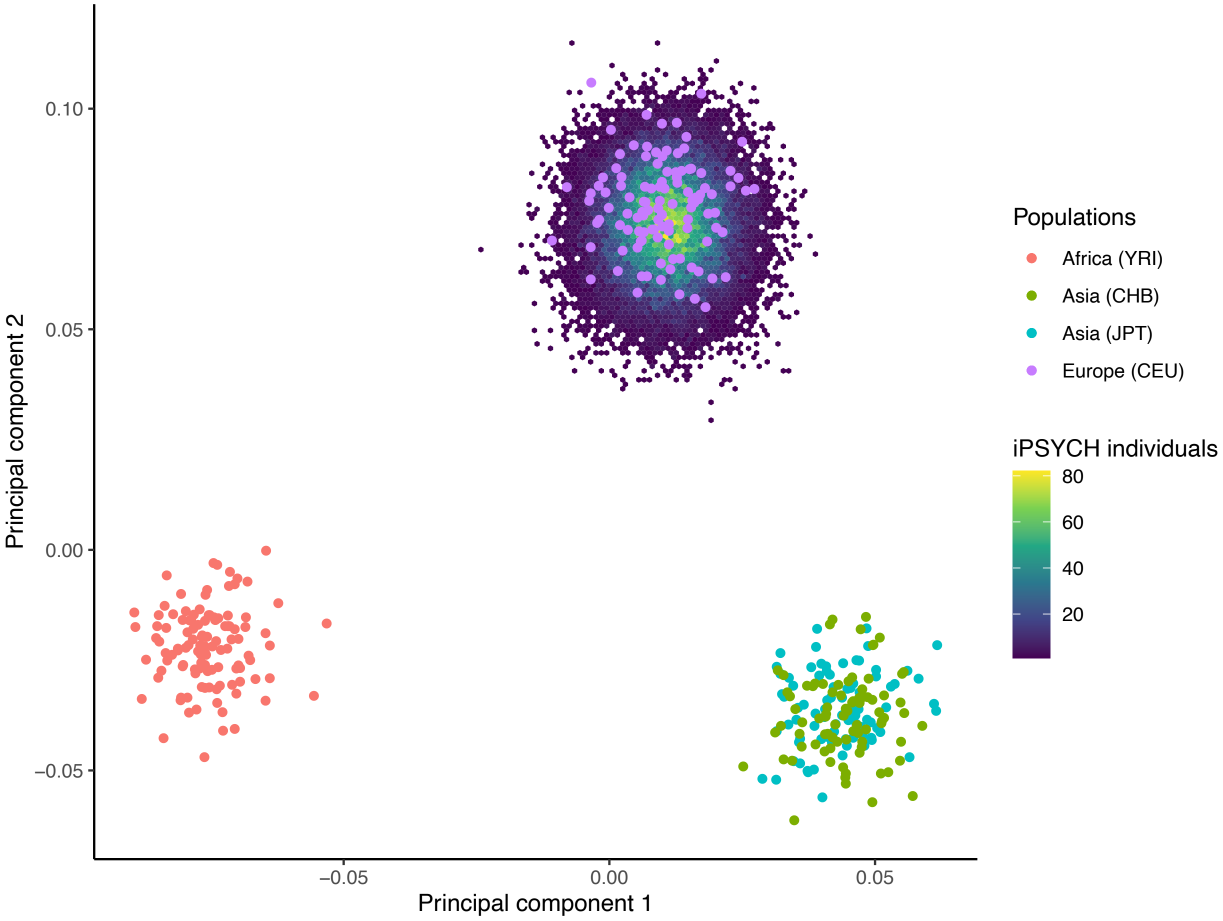

Fig. S9. Visualization of the population stratification by principal component analysis. Principal component analysis (PCA) plot showing the iPSYCH sample with available genotype data (31,863 individuals) together with part of the HapMap cohort including individuals of Japanese (JPT), Chinese (CHB), Yoruba (YRI) and European decent (CEU) on the 516 SNPs included in the study. The PCA was done using SmartPCA from the EIGENSOFT package.

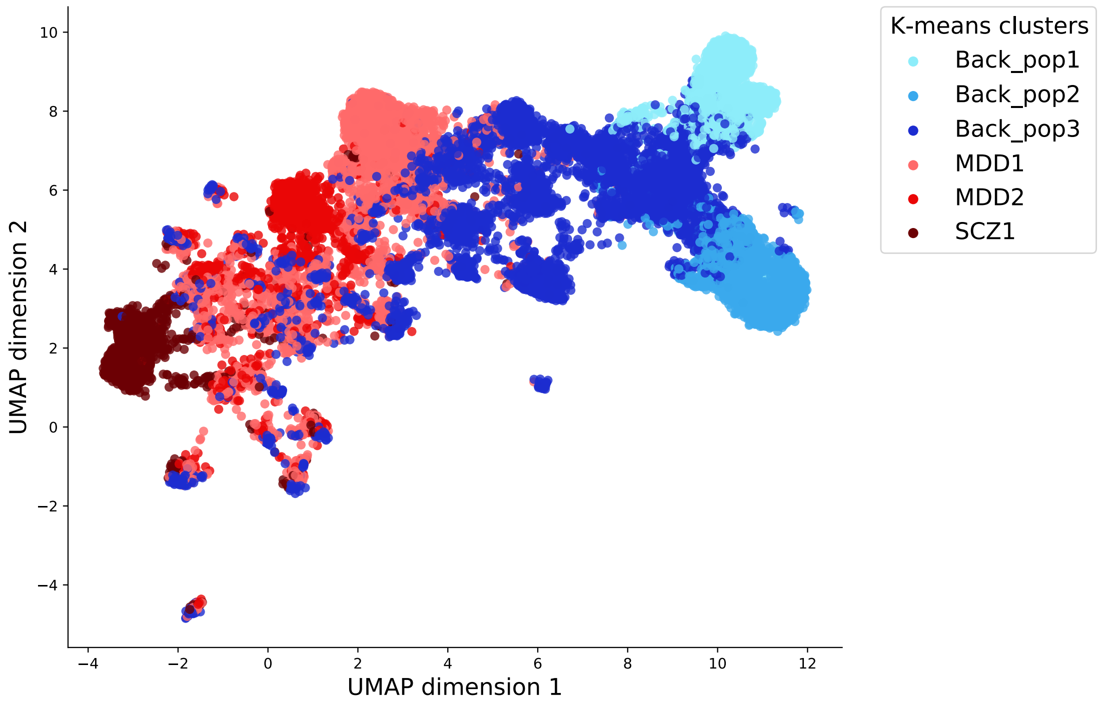

Fig. S10. Assessment of impact of missing data on clustering of the latent space.

Two-dimensional UMAP visualization of the latent representation of the data using the Variational Autoencoder (VAE) without individuals with missing genotype data due to failing one of the quality control step. Each dot represents a patient and for GDPR purposes we have masked all single occurrences not in proximity with other individuals to ensure privacy (less than 20 individuals were masked in total). The individuals are colored by the results of the K-means clustering of the latent representation.

	All individuals	Depression	Schizophrenia	Background population
**Number of individuals**	42103	15740	3896	22467
**Average age in 2016 [min,max]**	24 [10, 35]	27 [13, 35]	27 [13, 35]	22 [10, 35]
**Average age in prediction model (age masked) [min,max]**	21 [10, 35]	20 [10, 34]	21 [10, 34]	22 [10, 35]
**Sex (female) (%)**	23,896 (57%)	10,962 (70%)	1,986 (51%)	10,948 (49%)
**Any mental disorder (excl. MDD and SCZ)**	17,258 (41%)	11,651 (74%)	3,594 (92%)	2,292 (10%)
**Danish parental origin**	37,239 (88%)	14819 (94%)	3604 (93%)	18817 (84%)
**Danish or mixed Dansih parental origin**	40445 (96%)	15,705 (~100%)	3,883 (~100%)	20,857 (93%)
**Scandinavian or European parental origin (incl. DK)**	39,744 (94%)	15,449 (98%)	3798 (97%)	20497 (91%)
**Scandinavian or European parental origin (incl. DK + mixed)**	41,184 (98%)	15,740 (100%)	3,896 (100%)	21,548 (96%)
**Other parental origin**	919 (2%)	0 (0%)	0 (0%)	919 (4%)
**Average birth weight [95% CI]**	3,458g [3,453g, 3,464g]	3,414g [3,405g, 3,423g]	3,420g [3,402g, 3,439g]	3,497g [3,489g, 3,504g]
**Avgerage Gestational age [95% CI]**	39.56 weeks [39.54 weeks, 39.58 weeks]	39.62 weeks [39.60 weeks, 39.65 weeks]	39.53 weeks [39.46 weeks, 39.60 weeks]	39.52 weeks [39.49 weeks, 39.55 weeks]
**Average apgar score at 5 min [95% CI]**	9.86 [9.85, 9.86]	9.86 [9.85, 9.88]	9.84 [9.82, 9.87]	9.86 [9.85, 9.87]
**Missing genotype data (faild QC)**	4,106 (10%)	7 (0.04%)	5 (0.1%)	4,094 (18%)

Table S1.
Overview of basic descriptive statistics of the cohort for the individuals included as background population control or diagnosed with depression and/or schizophrenia.

Cluster	Depression	schizophrenia	background population
**A-Back_pop1 (VAE)**	0%	0%	100%
**A-Back_pop2 (VAE)**	0%	0%	100%
**A-Back_pop3 (VAE)**	6%	1%	93%
**A-MDD1 (VAE)**	100%	6%	0%
**A-MDD2 (VAE)**	100%	12%	0%
**A-SCZ1 (VAE)**	44%	99%	1%
**C1 (PCA)**	13%	4%	85%
**C2 (PCA)**	100%	8%	0%
**C3 (PCA)**	0%	0%	100%
**C4 (PCA)**	79%	15%	17%
**C5 (PCA)**	48%	100%	0%
**C6 (PCA)**	88%	17%	8%
**C1 (UMAP)**	87%	4%	13%
**C2 (UMAP)**	52%	83%	5%
**C3 (UMAP)**	10%	1%	90%
**C4 (UMAP)**	80%	13%	18%
**C5 (UMAP)**	87%	23%	3%
**C6 (UMAP)**	2%	0%	98%
**C1 (sPCA)**	47%	100%	0%
**C2 (sPCA)**	13%	4%	84%
**C3 (sPCA)**	15%	4%	83%
**C4 (sPCA)**	100%	8%	0%
**C5 (sPCA)**	100%	14%	0%
**C6 (sPCA)**	36%	5%	63%
**C1 (TSVD)**	12%	3%	86%
**C2 (TSVD)**	14%	4%	83%
**C3 (TSVD)**	95%	19%	0%
**C4 (TSVD)**	100%	14%	0%
**C5 (TSVD)**	100%	8%	0%
**C6 (TSVD)**	47%	100%	0%

Table S2.
Percent distribution of individuals included as background population control or diagnosed with depression and/or schizophrenia in each identified K-means cluster using either VAE, PCA, UMPA, sparse PCA (SPCA), or truncated singular value decomposition (TSVD) for dimensionality reduction.

Dataset	Analysis A	Analysis B	Analysis C
**Psychiatric disorders**	0.76	0.79	0.75
**Severity**	0.80	0.65	0.93
**Other medical conditions**	0.25	0.082	0.31
**Medical birth data (continuous)**	0.40	0.27	0.33
**Medical birth data (categorical)**	0.015	0.0094	0.11
**Genomics**	0.50	0.71	0.55
**HLA data**	0.21	0.0084	0.31
**Family diagnoses**	0.58	0.42	0.44

Table S3.

Impact of each data modality on the clustering when set to missing prior to VAE integration measured by the change in adjusted Rand Index. The change is calculated by the difference in adjusted Rand Index between the true labels from the VAE clustering on all data and the labels identified with the dataset set to missing.

Model	AUC	Cluster AUCs	MCC	Accuracy	By-chance accuracy
**Overall model**	0.81	Back_pop: 0.81<br>MDD: 0.74<br>SCZ: 0.65	0.39	65.0%	41.6%
**Model A**	0.83	A-Back_pop1: 0.97<br>A-Back_pop2: 0.94<br>A-Back_pop3: 0.81<br>A-MDD1: 0.61<br>A-MDD2: 0.63<br>A-SCZ1: 0.68	0.37	49.1%	21.3%
**Model B**	0.72	B-MDD1: 0.62<br>B-MDD2: 0.80<br>B-MDD3: 0.65<br>B-MDD4: 0.54<br>B-MDD5: 0.64<br>B-MDD6: 0.77<br>B-SCZ1: 0.66	0.18	31.4%	20.4%
**Model C**	0.79	C-SCZ1: 0.70<br>C-SCZ2: 0.71<br>C-SCZ3: 0.73<br>C-SCZ4: 0.80<br>C-SCZ5: 0.73<br>C-SCZ6: 0.86<br>C-SCZ7: 0.85	0.29	40.5%	15.3%

Table S4.

Overview of the AUC, Matthew Correlation Coefficient (MCC) and accuracy of each of the four prediction models of broad diagnostics of background population control group, depression (MDD) or schizophrenia (SCZ) as wells as the clusters identified in cluster analysis A, B and C. By chance accuracy is calculated as the combined weighted probability of identifying each cluster correctly $\sum_{i=1}^{k} x_i^2$ with x being the size of the cluster divided by the total number of individuals.

Dataset	Overall model	Model A	Model B	Model C
**Psychiatric disorders**	1.16%	1.64%	9.83%	23.21%
**Other medical conditions**	0.83%	3.45%	0.65%	1.41%
**Medical birth data**	9.51%	7.58%	1.37%	0.77%
**Genomics**	15.96%	24.07%	17.18%	8.08%
**HLA data**	0.066%	0.053%	0.25%	0.13%
**Family diagnoses**	24.37%	26.87%	18.99%	0.38%

Table S5.

Overview of the observed reduction in prediction accuracy of all models for each of the included dataset collections when setting all features to the same value (value for missing data) when evaluating the model on the test data.

Data S1. Detailed input data table (separate file).

Information on each input feature including feature name, feature description, ICD-10 and ICD-8 codes for the feature if applicable, feature encoding and information on which cluster analysis the feature was included in.

Data S2. Cluster signature confidence intervals and p-values for cluster analysis A (separate file).

Tab1: Confidence intervals of cluster signatures in analysis A.
Cluster signatures identified in cluster analysis A of background population control, depression (MDD) and schizophrenia (SCZ). For mental disorders we grouped all features into ICD-10 blocks within the F chapter, except mood disorders that are divided into single episode MDD, recurrent MDD and bipolar disorder. The scale illustrates the fraction of individuals with at least one diagnosis within the ICD-10 block for that cluster. Both severity and medical birth data are Z-score normalized showing from low to high values. For severity hospital contacts are a combined average of both days admitted and number of admissions. Family history and medical conditions are a combined count of the average number of occurrences across all included diagnoses per individual in the cluster. For each group the 95-% Confidence Intervals (CI) are reported.
Tab2: Adjusted P-values for cluster signatures in analysis A.
Significance level of cluster signatures identified in cluster analysis A of background population control, depression (MDD) and schizophrenia (SCZ). All p-values are adjusted for multiple testing.
Tab3: Confidence intervals of raw cluster signatures for in analysis A.
Cluster signatures of the raw input values for mental disorder severity and medical birth data identified in cluster analysis A of background population control, depression (MDD) and schizophrenia (SCZ). Information on data units can be found in Data S1.
Tab4: Adjusted p-values for all included SNPs and HLA alleles in analysis A.
Significance level of HLA alleles and individual SNP distributions as either homozygote for risk allele, heterozygote or homozygote for reference allele across clusters identified in cluster analysis A of background population control, depression (MDD) and schizophrenia (SCZ). All p-values are adjusted for multiple testing.

Data S3. Cluster signature confidence intervals and p-values for cluster analysis B (separate file).

Tab1: Confidence intervals of cluster signatures in analysis B.
Cluster signatures identified in cluster analysis B of depression (MDD) and schizophrenia (SCZ). For mental disorders we grouped all features into ICD-10 blocks within the F chapter, except mood disorders that are divided into single episode MDD, recurrent MDD and bipolar disorder. The scale illustrates the fraction of individuals with at least one diagnosis within the ICD-10 block for that cluster. Both severity and medical birth data are Z-score normalized showing from low to high values. For severity hospital contacts are a combined average of both days admitted and number of admissions. Family history and medical conditions are a combined count of the average number of occurrences across all included diagnoses per individual in the cluster. For each group the 95-% Confidence Intervals (CI) are reported.
Tab2: Adjusted P-values for cluster signatures in analysis B.
Significance level of cluster signatures identified in cluster analysis B of depression (MDD) and schizophrenia (SCZ). All p-values are adjusted for multiple testing.
Tab3: Confidence intervals of raw cluster signatures for in analysis B.
Cluster signatures of the raw input values for mental disorder severity and medical birth data identified in cluster analysis B of depression (MDD) and schizophrenia (SCZ). Information on data units can be found in Data S1.
Tab4: Adjusted p-values for all included SNPs and HLA alleles in analysis B.
Significance level of individual SNP distributions as either homozygote for risk allele, heterozygote or homozygote for reference allele across clusters identified in cluster analysis B of depression (MDD) and schizophrenia (SCZ). All p-values are adjusted for multiple testing.

Data S4. Cluster signature confidence intervals and p-values for cluster analysis C (separate file).

Tab1: Confidence intervals of cluster signatures in analysis C.
Cluster signatures identified in cluster analysis C of schizophrenia (SCZ). For mental disorders we grouped all features into ICD-10 blocks within the F chapter, except mood disorders that are divided into single episode MDD, recurrent MDD and bipolar disorder. The scale illustrates the fraction of individuals with at least one diagnosis within the ICD-10 block for that cluster. Both severity and medical birth data are Z-score normalized showing from low to high values. For severity hospital contacts are a combined average of both days admitted and number of admissions. Family history and medical conditions are a combined count of the average number of occurrences across all included diagnoses per individual in the cluster. For each group the 95-% Confidence Intervals (CI) are reported.
Tab2: Adjusted P-values for cluster signatures in analysis C.
Significance level of cluster signatures identified in cluster analysis C of schizophrenia (SCZ). All p-values are adjusted for multiple testing.
Tab3: Confidence intervals of raw cluster signatures for in analysis C.
Cluster signatures of the raw input values for mental disorder severity and medical birth data identified in cluster analysis C of schizophrenia (SCZ). Information on data units can be found in Data S1.
Tab4: Adjusted p-values for all included SNPs and HLA alleles in analysis C.
Significance level of individual SNP distributions as either homozygote for risk allele, heterozygote or homozygote for reference allele across clusters identified in cluster analysis C of schizophrenia (SCZ). All p-values are adjusted for multiple testing.
